# Natural Killer Cells—An Epigenetic Perspective of Development and Regulation

**DOI:** 10.3390/ijms17030326

**Published:** 2016-03-01

**Authors:** Alexander Schenk, Wilhelm Bloch, Philipp Zimmer

**Affiliations:** Department of Molecular and Cellular Sports Medicine, Institute for Cardiovascular Research and Sports Medicine, German Sport University Cologne, Am Sportpark Muengersdorf 6, 50933 Cologne, Germany; a.schenk@dshs-koeln.de (A.S.); w.bloch@dshs-koeln.de (W.B.)

**Keywords:** epigenetic, methylation, histone, miRNA, NK cell, natural killer cell, lymphocytes, exercise

## Abstract

Based on their ability to recognize and eliminate various endo- and exogenous pathogens as well as pathological alterations, Natural Killer (NK) cells represent an important part of the cellular innate immune system. Although the knowledge about their function is growing, little is known about their development and regulation on the molecular level. Research of the past decade suggests that modifications of the chromatin, which do not affect the base sequence of the DNA, also known as epigenetic alterations, are strongly involved in these processes. Here, the impact of epigenetic modifications on the development as well as the expression of important activating and inhibiting NK-cell receptors and their effector function is reviewed. Furthermore, external stimuli such as physical activity and their influence on the epigenetic level are discussed.

## 1. Natural Killer Cells

Natural killer cells (NK cells) are historically named by their ability to kill target cells without prior priming on a “natural” way [1]. As a part of the innate cellular immune system, they are able to recognize and eliminate tumor- and virus-infected cells, parasites as well as certain types of bacteria. NK cells liquidate their targets by releasing a variety of cytokines, such as interferon (IFN)-γ, tumor growth factor (TGF)-β and IL-10 [2,3,4], and by secreting toxic agents like perforine and granzyme B. IFN-γ secretion enhances the NK cell activity and regulates the innate and adaptive immune system by stimulating macrophages or enhancing the cytotoxicity of CD8+ T-lymphocytes [5,6]. Both TGF-β and IL-10 are secreted as immune regulators with immune suppressive properties [6].

Their phenotype is determined by surface expression of the NK cell marker CD56 and a concomitant absence of the T-cell marker CD3 (CD3^−^ CD56^+^) [1]. In general NK cells are divided in a CD56^bright^ and a CD56^dim^ subgroup. CD56^bright^ NK cells express high amount of the NK cell marker CD56 and are characterized by a lower cytotoxic capacity but high secretion of cytokines upon stimulation [1,2]. CD56^bright^ NK cells represent the minority of NK cells and are mainly located in secondary lymphoid tissues [1,7]. In contrast, CD56^dim^ NK cells express CD56 in low amounts on the cell surface and display a high cytotoxic capacity [1,2]. With about 90%, CD56^dim^ are the majority of NK cells circulating through the body [1,7].

Current literature suggests a third subgroup of NK cells, described as memory NK cells. This population of CD57^+^ NKG2C^hi^ NK cells has adaptive functions and mediates a fast recurring response against viral infections like Cytomegalo Virus (CMV) [8].

The effector function of NK cells is regulated by an orchestra of activating and inhibitory receptors on the cell surface. These receptors are encoded by the germ line and recognize structures of high molecular weight [9]. All inhibitory receptors contain a tyrosine-based inhibitory motif (ITIM) in their cytoplasmatic tail and promote their signaling in a major histocompatibility complex (MHC) class I-mediated or independent manner [10]. Receptors with MHC class I-mediated signaling are inhibitory members of the killer cell immunoglobulin-like receptors (KIR), leukocyte immunoglobulin-like receptors or C-type lectin receptors. MHC class I-independent signaling is mediated by single receptors like the killer cell lectin-like receptor G1 (KLRG1) or the NK-cell-receptor protein 1 (NKR-P1). In contrast to the inhibitory receptors, signaling of activating receptors is mediated by the association with adaptor proteins containing an immunodominant tyrosine based activation motif (ITAM) on their cytoplasmic tail, like DAP10 or FcεRIγ [10]. NKG2D, CD16 and the natural cytotoxicity receptors (NCRs) are described as the most important activating NK cell receptors in humans.

The activation of NK cells effector function is mediated by a critical balance of signals from inhibitory and activating receptors. When an activation of effector function is achieved, NK cells secrete granules containing effector molecules like perforin and granzyme B. Perforin is a 70 kDa protein containing a conserved membrane attack complex of Complement/Perforin (MACPF) domain [11]. The MACPF domain is made up for pore-forming proteins and is further required for the synchronized polymerization of perforin proteins into the lipid bilayer of membranes. Upon the release of perforin, the proteins suffer a conformational change and polymerize to build pores in the cell membrane of target cells [11], whereby the membrane integrity of the target cells gets lost. The second effector molecule, granzyme B, is a serine protease with similarities to the apoptotic cysteine protease family (caspase) [11]. It induces DNA damage by direct and indirect procaspase activation and initiates cell death of the target cells [12].

## 2. Epigenetic Modifications

In 1942, about 20 years before the Nobel-Prize for defining the ultrastructure of the DNA was awarded, Conrad Waddington defined epigenetic as “The branch of biology which studies the causal interactions between genes and their products, which bring the phenotype into being”. Newer definitions of epigenetic describe it as heredity which is independent of the DNA sequence and as research of changes in gene expression and mitotic heredity of gene expression patterns [13]. The actual comprehension of epigenetic is the regulation of genomic activity leading to a functional genome. Epigenetic modifications include chromatin modulating processes such as DNA methylation or posttranslational histone modifications (e.g., phosphorylation, methylation or acetylation) as well as the expression of little non-coding RNA molecules (microRNA) [14].

DNA methylation is catalyzed by DNA-methyl-transferases (DNMTs) by the addition of a methyl-group on cytosines in the 5th position. Therefore, DNA methylation occurs at cytosine-guanine-dinucleotides (CpGs) and mostly in regions with a high CpG density. At the beginning of epigenetic, research DNA methylation was described to be inhibitory for gene expression. It is described that methylation of CpG rich regions could inhibit the binding of transcription factors that recognize these regions [15] and further proteins that recognize and bind methylated DNA sequences would inhibit the transcription machinery [16]. Nowadays, is known that DNA methylation could achieve both, inhibit gene transcription or enhance it, depending on the location within the gene. Whether DNA methylation within the promoter region is inhibitory, DNA methylation in the gene body could promote gene transcription [17].

Histone modifications result in opening or closing the chromatin structure. Depending on the posttranslational modification, repression or activation of gene transcription is achieved. For example, the acetylation of lysine residues on histone 3 and 4 is associated with active transcription, whether methylation of lysine residues on these histones could be activating or inhibitory [18]. The most prominent histone modification is the acetylation of lysine residues. The level of histone acetylation is established by the interplay between histone acetyltransferases (HAT, acetylating) and histone deacetylases (HDAC, deacetylating) [19]. The acetylation of lysine residues neutralizes the positive charge of histone tails and decreases the affinity to the negatively charged DNA backbone and therefore opens the chromatin structure and favoring active transcription [19].

Finally, the expression of microRNA molecules is described as epigenetic mechanism. These small endogenous, single-stranded RNA molecules modulate gene expression by binding to complementary sites in the 3′-untranslated region (3′-UTR) of the target genes mRNA [4]. This interaction promotes the degradation of mRNA and can further inhibit the translational process. The expression of microRNA itself could also be regulated by the epigenetic modifications mentioned above.

## 3. Epigenetic Modifications in NK Cell Development

Only a few studies have investigated the influence of epigenetic modifications on the differentiation and maturation of NK cells. Santourlidis *et al.* [20] showed that KIR gene expression is suppressed by DNA methylation in hemopoietic progenitor cells (HPCs) and KIR^−^ Lymphocytes. In contrast, NK cells and other KIR expressing Lymphocytes reveal a demethylation of KIR genes that resulted in the abolishment of KIR gene expression. Similar results were reported by Gao *et al.* [21], who described the DNA demethylation as a process, leading to a specific profile of KIR expression. The authors depicted the process of DNA demethylation to be initiated by opening of chromatin structure. The chromatin remains open for expressed KIR genes, while non-expressed genes demonstrate condensed chromatin structures. Therefore, the authors state bivalent chromatin accessibility within one gene locus. Besides chromatin modifications, Cichocki *et al.* [22] found the miRNA miR-181 to be an important regulatory element in the development of NK cells. This was shown by a knockdown of miR-181, which was associated with a decreased differentiation of HPCs to mature NK cells, whereas an over-expression of miR-181 led to an increased differentiation into NK cells [22]. Furthermore, there is an increasing amount of miR-181 in NK progenitor cells with increasing stage of differentiation [22]. MiR-181 influences the NK cell differentiation by its target, the Nemo-like kinase which down regulates Notch signaling [22]. Notch signaling seems to be essential during the maturation of NK cells [23,24].

Regarding epigenetic modifications during differentiation and maturation, most studies focused on memory NK cells which display elements of the adaptive immune system and are induced by an infection with cytomegalovirus (CMV). Memory NK cells have been discovered in mouse models and have been described to have properties of memory T cells, such as being self-renewing, long-lived and to show expansion upon a second viral challenge [25]. These cells are described as CD57^+^ and NKG2C^hi^ [8] as well as deficiency for the FcRγ, the signal molecules SYK, DAB2, EAT-2, and the transcription factors PLZF and IKZF2 [26]. Deficiency for FcRγ and EAT-2 has been shown to be dependent on the promoter methylation in these genes [27], which is indicated by a hypermethylation of CpG sites in their promoter region. Lee and colleagues [26] showed similar results for SYK.

Furthermore, the DNA methylation profile of canonical and memory NK cells was compared to CD8^+^ and CD4^+^ T cells, what resulted in higher similarities between memory NK cells and CD8^+^ and CD4^+^ T cells than between memory and canonical NK cells [8,27]. The comparison of memory and canonical NK cells revealed 2372 differentially methylated regions (DMRs), whereas the memory NK cells and CD8+ T cells differ in just 61 DMRs [27]. Imprinting of the IFN-γ promoter is reported to be similar in memory NK cells and memory T_H_1 cells [8]. Therefore, the authors suggested that the development of memory NK cells is similar to that of T cells, whereby the adaptive functions of memory NK cells may be explained. However, there is still lack of information in which state of NK cell development the switch into a differentiation to memory NK cells appears, or if canonical matures NK cells develop to memory NK cells.

## 4. Epigenetic Regulation of NK Effector Function

As mentioned above, NK cell activity is related to a complex interaction of activating and inhibiting receptors on the NK cell surface. Interestingly, both activation patterns (cytokines or secretion of cytotoxic agents) seem to be epigenetically regulated.

Incubation with HDAC inhibitors (HDACi) like suberoylanilide hydroxamic acid (SAHA) or valproic acid (VPA) suppresses Interleukin (IL)-2-activated NK cell cytotoxicity [28]. These results have been improved by Fernandes-Sanchez *et al.* [19] who found an impaired NKG2D dependent degranulation and IFN-γ secretion after HAT incubation. Furthermore, the authors showed that NKG2D expression is depending on histone acetylation.

Besides histone modifications, the DNA methylation is important to orchestra the NK cell effector function. To investigate the effect of DNA methylation, demethylating agents like 5-azacytidine (Aza) and decitabine (Deci) are used. Aza is an analogue to the DNA base cytidine, whereas Deci is the analogue to desoxycytosine. Schmiedel *et al.* [29] described two different modes of action for these agents. In the absence of proliferation is Aza considered to impair the synthesis of mRNA, whereas Deci promotes gene transcription. In line with these assumptions, the trials revealed a suppression of NK cell function by Aza [21,29] and an enhanced function by Deci [29]. Both the cytotoxicity and the production of IFN-γ upon contact to target cells were affected by the two demethylating agents [21,29]. The suppression of cytotoxicity by treatment with Aza was shown to be related to impaired functional parameters such as the release of the effector molecules perforin and granzyme B [21].

It has to be noted, that IL-2 seems to be a cofactor for DNA demethylating or histone acetylating agents. While Gao *et al.* [21] and Schmiedel *et al.* [29] used IL-2 in the cultivation of NK cells, Ogbomo *et al.* [28] tried the cultivation with and without IL-2. Cultivation with IL-2 resulted in the described influences of epigenetic modifiers. In contrast, this influence abolished when the cells were cultivated in the absence of IL-2. This was explained in the activating effect of IL-2 on NK cells that results in a three to four fold higher expression of activating and inhibitory receptors on the cell surface [28], whereby the influence of the epigenetic modifiers is more pronounced.

## 5. Epigenetic Regulation of NK Cell Receptors

As mentioned above, epigenetic modifications are involved in NK cell differentiation and regulation of effector function. NK cell receptors are differentially expressed through the development of HPCs to mature NK cells and are crucially involved in the induction and inhibition of NK cell effector function.

The suppression of NK cell cytotoxicity by incubation with HDACi observed by Ogbomo *et al.* [28] was due to a reduction of transcription and surface expression of the activating receptors NKp30 and NKp36. Beside these two receptors, the authors could not find influences of HDACi incubation on the activating receptors NKp44, NKG2D and DNAM-1 as well as inhibitory KIR and the inhibitory NKG2A. These findings suggest an exclusive regulation of NKp30 and NKp46 by histone acetylation.

### 5.1. Killer Cell Immunoglobin-Like Receptors (KIR)

KIR belong to the Ig superfamily and are named by the number of extracellular immunoglobulin domains (e.g., 2D) and their type of signaling, which can be stimulatory (S) or inhibitory (L) [30].

Gao *et al.* [21] investigated the promoter methylation of CpG islands of KIR2DL1, KIR2DL2/L3 as well as of the KIR3DL1 gene. They showed a dense methylation pattern in these gene regions in untreated NK-92 MI cells that resulted in a very low presence of these molecules on the cell surface. After treatment with the demethylating compound 5-Aza surface, expression of KIR genes has been induced [21,31]. Furthermore, an investigation of KIR3DL1^+^ and KIR3DL1^−^ NK cells showed a densely methylated KIR3DL1 promoter in KIR3DL1^−^ cells and a completely unmethylated promoter in KIR3DL1^+^ cells [21,32]. These findings are in line with the results of Santourlidis *et al.* [31] who revealed that non expressed KIR genes have been hypermethylated (CpG methylation of 70%–100%), whereas expressed KIR genes have been hypomethylated (CpG methylation < 70%). Furthermore, the authors stated that inhibition of a single KIR gene expression is not restricted to the methylation of specific CpG sites, but rather due to the methylation of CpG sites throughout the KIR gene. One exception in KIR gene regulation is KIR2DL4, which is constitutively expressed [3] and not affected by DNA demethylating or histone acetylating treatments [31].

During the development of HPCs to mature NK cells, the DNA demethylation of KIR genes leads to KIR expression. But DNA methylation does not just determine which KIR gene is expressed, it also determines which allele expresses the KIR gene. KIR genes are mostly expressed in a mono-allelic manner with non-expressed alleles exhibit DNA hypermethylation and expressed alleles DNA hypomethylation [3]. Furthermore Chan *et al.* [3] mentioned that the expression of a KIR gene does not determine whether another KIR gene is expressed from the same, the other or both alleles. Notably, the allele specific restriction of gene transcription remains stable. 

Besides chromatin modifications, KIR genes are also regulated by microRNA. As reported by Davies *et al.* [33], KIR genes have a proximal promoter that features bidirectional promoter activity. The authors found an 88 bp fragment of the bidirectional distal promoter of the KIR3DL1 gene that possesses 100% similar sequence with the promoters of KIR2DL1, KIR2DS1, KIR2DL2, KIR2DS2, KIR3DS1 and KIR3DL2. Upon induction of the promoter in the forward direction, coding transcripts of the KIR genes are generates, whereas the induction in the reverse direction leads to antisense transcripts [33].

Cichockie *et al.* [32] described a 28 base PIWI-like RNA which induces a 90% reduction in KIR expression. This reduction is explained by binding of the antisense transcript to the mRNA and an inhibition of translation respectively degradation of the double stranded RNA. Furthermore, there might be a local interaction of this PIWI-like RNA with chromatin modifying enzymes that shut down single KIR genes [32].

In contrast to DNA methylation, histone acetylation seems to play a less important role in KIR gene regulation. Incubation of NK cells with HDACi did not affect KIR gene expression [28,31]. Moreover, KIR genes exhibit highly similar histone acetylation signatures, which are typically found in expressed genes. This fact puts the KIR genes into a state of readiness for transcription which is depending on the DNA methylation as critical epigenetic modification in the regulation of KIR gene expression [20,31].

### 5.2. NKG2D

In addition to KIR gene regulation, epigenetic modifications have been reported to be involved in the expression of NKG2D, which is one the most important activating NK cell receptor. Fernandes-Sanchez *et al.* [19] found a regulation of NKG2D expression by DNA demethylation and histone modifications. Thereby, active transcription has been characterized by high levels of H3K9Ac and reduced repressive histone marks, like trimethylation at H3K27 [19]. Further, an incubation of NK cells with a HAT inhibitor (HATi) resulted in a downregulation of NKG2D expression on the cell surface by reduction of H3K9Ac, resulting in reduced cytotoxic capacity of NK cells [19]. Furthermore, DNA methylation has been shown to be involved in the regulation of NKG2D expression. Whether NKG2D non-expressing cells show DNA hypermethylation of the NKG2D gene, or this gene was partial or full demethylated in NKG2D expressing cells [19], is unknown.

**Figure 1:**
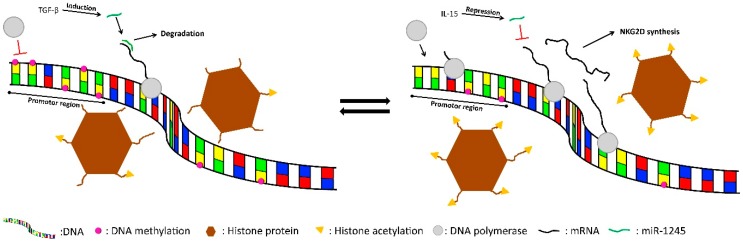
Epigenetic regulation of NKG2D. DNA methylation in the promoter region and deacetylated histone 3 lysine 9 (H3K9) suppress gene expression. TGF-β induces miR-1245 leading to a degradation of NKG2D mRNA. If DNA is demethylated and histones are acetylated, gene expression is more pronounced. IL-15, an inducer of NKG2D expression, represses miR-1245 synthesis.

Besides histone modifications and DNA methylation, microRNAs are involved in the regulation of NKG2D expression. Espinoza *et al.* [4] have showed the 3′untranslated region (3′UTR) of the NKG2D gene to contain a target site for the microRNA miR-1245. The authors demonstrate this regulation and the interaction of miR-1245 with the NKG2D 3′UTR by over-expression of miR-1245 what resulted in a down regulation of NKG2D expression and impairment of NKG2D dependent immune functions, including cytotoxicity and cytokine secretion. The expression of miR-1245 is influenced by the cytokines IL-15 and TGF-β1. IL-15 is a potent inducer of NKG2D expression. Binding of IL-15 leads to down regulation of miR-1245, whereas TGF-β1 is a repressor of NK cell function and binging of TGF-β1 induces miR-1245 expression [4]. Interestingly, the number of primary transcripts of miR-1245 does not change, while the number of mature miR-1245 is altered by posttranscriptional processing [4]. This implicates that miR-1245 is strongly involved in acute regulation of NKG2D. In addition, Espinoza *et al.* [4] described miR-1245 as an endogenous autoregulatory mechanism to maintain NKG2D expression at physiological levels and further to take part in the negative feedback of TGF-β1 which is secreted by stimulated NK cells, for the prevention of overstimulation. NKG2D regulation is illustrated in Figure 1.

## 6. External Stimuli

Life style factors such as physical (in-) activity and nutrition are supposed to influence the immune system and its function [34]. NK cells are sensitive to exercise regarding their number and distribution in the blood, as well as their NKCA (reviewed by Gleeson *et al.* [35]).

Since physical activity and exercise are known to induce short- and long-term epigenetic alterations in various cell types and tissues [36,37,38,39,40], one could speculate that NK cells are also affected by similar mechanisms. The first study in this context was conducted by Nakajima *et al.* [41] who have showed, that a six month endurance exercise program counteracts an age dependent promotor DNA demethylation of the ASC gene in peripheral blood monocytes which encodes for pro-inflammatory cytokines. Since epigenetic modification can be cell specific, such results are hard to transfer to single subsets of immune cells.

Against this background, Zimmer *et al.* focused on changes in histone acetylation and DNA methylation in NK cells in response to exercise. In a first study, the influence of chemotherapeutic treatment and a single bout of exercise on global H3K9Ac and H4K5Ac in NK cells of B-cell non-hodgkin lymphoma patients and a healthy control group has been determined [42]. After treatment, patients revealed a reduced H3K9Ac compared to healthy controls. Furthermore, a correlation between H4K5Ac and endurance capacity has been detected [42], which links the physical activity to NK cell regulation. However, neither in patients nor in controls histone acetylation in NK cells has been affected by a single bout of low to moderate endurance exercise. In contrast, a prolonged and more intense single bout of exercise (half-marathon) resulted in a significant increase in global H4K5ac in both, cancer patients in the aftercare as well as in healthy controls [43]. Interestingly, this epigenetic alteration, which has been most pronounced 24 h after the run, has beenaccompanied by an elevated expression of NKG2D. Although global DNA methylation has been unaltered in this interventional study, the authors state that gene specific alterations could not be ruled out. These results are in line with those of Fernandes-Sanchez *et al.* [19] who demonstrated that NKG2D expression is regulated by histone modifications and DNA demethylation.

Another mechanism by which regular physical activity may alter the epigenome of NK cells is a reduction of psychophysiological stress which is associated with the secretion of glucocorticoids [44]. As reviewed by Shoneveld and Cidlowski [45], glucocorticoids lead to a functional impairment of the immune system which is at least partially driven by an altered gene expression. It is supposed that these changes are due to epigenetic modifications. As mediator of stress, the synthetic glucocorticoid dexamethasone (Dexa) has been used to test this hypothesis.

Krukowski *et al.* [46] revealed a decreased histone acetylation at the IFN-γ promotor, which is accompanied by a reduced accessibility and expression of this cytokine after treatment with Dexa. Busch *et al.* [47] confirmed these results and could further show a decrease of global histone acetylation as well as a reduced acetylation of histone 4 lysine position 8 (H4K8). In contrast, cultivated NK cells which have been treated with Dexa in the presence of IL-2 have showed no alterations in histone acetylation at the IFN-γ promoter [48]. Therefore, the authors stimulated the cultivated NK cells with IL-12 and found more histone acetylation at the IFN-γ enhancer as well as higher INF-γ mRNA and protein levels. Similar results have been found for IL-6. Eddy *et al.* [48] and concluded that the Dexa treatment increases the histone acetylation, which leads to a higher chromatin accessibility in proinflammatory gene regions and contributes to an enhanced proinflammatory cytokine production after cellular stimulation. However, it is unclear why the IL-2 substitution alters the influence of the Dexa treatment. Since IL-2 is a potential stimulator of IFN-γ production [6], it might cover the influence of Dexa on the IFN-γ gene.

All studies mentioned above also analyzed the histone acetylation at the promoter regions of perforin and granzym B and could show that treatment with Dexa resulted in a reduced histone acetylation [46,47,48] which is displayed by reduced mRNA and protein levels as well as a reduction in NKCA [48]. These results indicate that the impact of the Dexa treatment is independent of IL-2 stimulation and decreases the basal expression of the effector molecules.

## 7. Conclusions

Epigenetic modifications seem to play a key role in NK cell development and regulation. On the one hand, a stable epigenetic imprinting is used for maintaining the way of differentiation. On the other hand, the dynamic properties of epigenetic modifications are used for adapting/reacting on internal and external stimuli (e.g., physical exercise). However, the significance of the described findings is limited by study designs. Although human NK-cells were frequently used, in most cases treatment took place in *ex vivo* experiments. Therefore, functional alterations of NK-cells which were detected in such experiments are hard to transfer from bench to practice. Translational research approaches combining basic research findings with clinical outcomes (e.g., prognosis, tumor size) are necessary to approve results from *ex vivo* experiments. Further research is needed to fulfill the understanding of the epigenetic regulation of NK cells and potential therapeutic implications.
